# Health Coverage and Financial Protection in Uganda: A Political Economy Perspective

**DOI:** 10.34172/ijhpm.2021.116

**Published:** 2021-08-29

**Authors:** Maria Nannini, Mario Biggeri, Giovanni Putoto

**Affiliations:** ^1^Department of Economics and Management, University of Florence, Florence, Italy.; ^2^Doctors with Africa CUAMM, Padova, Italy.

**Keywords:** Universal Health Coverage, Political Economy, Health Financing, Financial Protection, Uganda

## Abstract

**Background:** As countries health financing policies are expected to support progress towards universal health coverage (UHC), an analysis of these policies is particularly relevant in low- and middle-income countries (LMICs). In 2001, the government of Uganda abolished user-fees to improve accessibility to health services for the population. However, after almost 20 years, the incidence of catastrophic health expenditures is still very high, and the health financing system does not provide a pooled prepayment scheme at national level such as an integrated health insurance scheme. This article aims at analysing the Ugandan experience of health financing reforms with a specific focus on financial protection. Financial protection represents a key pillar of UHC and has been central to health systems reforms even before the launch of the UHC definition.

**Methods:** The qualitative study adopts a political economy perspective and it is based on a desk review of relevant documents and a multi-level stakeholder analysis based on 60 key informant interviews (KIIs) in the health sector.

**Results:** We find that the current political situation is not yet conducive for implementing a UHC system with widespread financial protection: dominant interests and ideologies do not create a net incentive to implement a comprehensive scheme for this purpose. The health financing landscape remains extremely fragmented, and community-based initiatives to improve health coverage are not supported by a clear government stewardship.

**Conclusion:** By examining the negotiation process for health financing reforms through a political economy perspective, this article intends to advance the debate about politically-tenable strategies for achieving UHC and widespread financial protection for the population in LMICs.

## Background

**Key Messages**

** Implications for policy makers**

- A political economy perspective is relevant to explain the evolution of health financing reforms and needs to be taken into account when pursuing universal health coverage (UHC).
- In Uganda political economy conditions are not yet favourable for universal coverage; in particular, interests and ideas are not conducive for expanding financial protection.
- Stewardship from the central government is essential to improve financial protection and more efforts are needed to ensure a major commitment for public health financing.

** Implications for the public**

Considering the influence of political economy on health financing reforms allows to disentangle the country-specific experience related to financial protection for universal health coverage (UHC). In Uganda, the role played by the central government and other stakeholders determines the current level of financial protection for the population. This research helps to identify the major obstacles against the implementation of health financing reforms towards UHC; furthermore, potential opportunities to improve the population coverage and financial protection are indicated. The adoption of a political economy perspective is relevant to enhance the understanding on the main processes shaping progress towards UHC and the usefulness of applying this analytical lens goes beyond the single case study of Uganda. It will be important, thus, to utilise political economy frameworks such as the one presented here as key to interpret the experiences of different low- and middle-income countries (LMICs).

Forty years after the Alma Ata declaration, the international community reaffirmed its commitment to ensure access to quality healthcare for the population of all countries. Universal health coverage (UHC), defined as a situation where people who need health services receive them without undue financial hardship, received renewed attention at the global level and was embraced in the Sustainable Development Goals.^1^ The objective of UHC is informed by a horizontal approach for system-level interventions and, thus, brings about important implications for low- and middle-income countries (LMICs).^2,3^ As part of the 2030 Agenda, international institutions strongly support the implementation of efficient and equitable health sector reforms for quality care, claiming, in particular, to ensure adequate financial protection for the population against the risk of financial catastrophe due to health expenditures.^4^

 An extensive literature^5-7^ investigates the main technical factors enabling LMICs to move towards UHC by enhancing health financing systems. Many studies^1,8-10^ argue for more systematic reforms to overcome the excessive fragmentation of health systems in LMICs. They point to the importance of a general growth in health spending and claim that the increase in health expenditure should be financed domestically.^1,5,11^ To address demand-side barriers to the utilization of health services, pre-payment financing strategies that avoid catastrophic expenditures for the population are strongly recommended.^12,13^ Although the discussion of the major technical approaches facilitating the expansion of health coverage is relevant, political determinants driving these improvements deserve more attention.^14^ Several authors indicate that a political economy perspective can contribute to understanding contingent paths to UHC.^15-17^ Health system analyses need to be supplemented with approaches that focus on the political dynamics surrounding reforms, as reflected in many studies.^2,18-25^

 Following this strand of literature, the present investigation advances the debate on the political economy of UHC by considering the case of Uganda and the country’s experience of health financing reforms. Our analysis identifies the effects of stakeholders’ interests and ideas on the negotiation process behind these reforms, and the resulting implications in terms of financial protection enjoyed by the population. A political economy framework is developed and tested in order to disentangle the Ugandan experience. The framework represents a preliminary output of the research, and it is functional to examine the different spheres which play a role in the political economy process. The investigation follows the line of reasoning presented in the framework and it is informed by a desk review, and 60 key informant interviews (KIIs) with major stakeholders in the health sector (32 at the national level and 28 in one district). The analysis focuses on the last two decades (starting from 2000) and investigates, in particular, the abolition of user-fees in public health facilities and the debate on National Health Insurance as two important cases of health financing reforms with relevant implications for financial protection.

###  Conceptual Framework

 Making progress towards UHC requires the convergence of several factors.^19^ In order to develop a coherent analysis of the Uganda’s experience of health financing reforms, we adopt a political economy framework inspired by existing knowledge about the politics sphere and UHC.^23^ Since many different theories have been used to interpret health reforms and underlying political processes, we draw on contributions from several authors in political economy and public health analysis. As noted by Fox and Reich,^14^ progress or delay in achieving positive health coverage outcomes strongly depends on the political economy discourse affecting the health system. Indeed, “countries moving towards UHC face a number of choices, from policy negotiations and decisions to financing and implementation, that are inherently political.”^7^ In this article, we refer to health policy as a public strategic plan of action to make progress towards the goal of UHC.^25^ Practical difficulties of implementing health policy within a specific national context reflect the complexity of politics, that here is related to “managing actors, organizations and institutions that have a stake in health reform.”^25^

 The processes driving policy design and policy-making for health financing reforms in LMICs is conceptualized in Figure. The circular and dynamic feature of the framework indicates the incremental nature of the process: the spheres of politics and policy are animated by stakeholders’ interactions and result in health coverage outcomes when policies are effectively implemented after the negotiation.^26^ In this sense, policy is a product of, and constructed through, political processes of negotiation where ideas, knowledge, interests, power and institutions are influential.^14^

**Figure:**
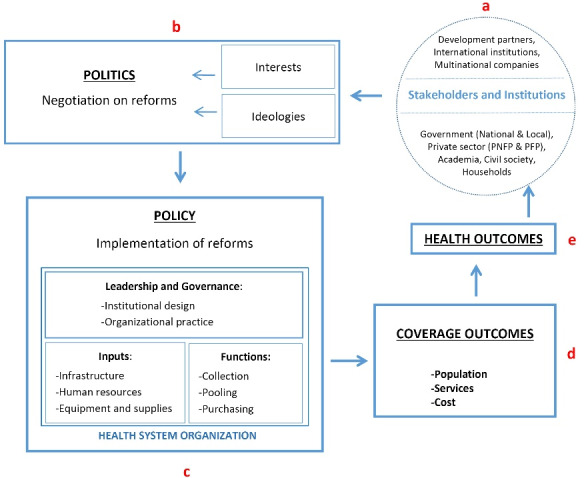
The Political Economy of Health Financing Reforms.

 The main actors behind the negotiation process on health reforms (“a” in Figure) refer to political institutions and public bureaucracies as well as non-state actors^15^ such as private sector, international agencies, civil society organisations and the academia. A country’s experience of reforms for UHC and, specifically, for health financing, is largely affected by the role played by the central government^12^; in this respect, the degree of consensus governments manage to build for the reform process is crucial,^26^ as well as the political commitment to allocate considerable resources to the health system.^27^ Within the public sector, visions on policy-making are often plural: finance ministries and health ministries who discuss the design of reforms may have conflicting priorities.^14^ Furthermore, external donors can greatly influence health system infrastructure; in case where they bypass the public sector, they may end up creating an unregulated private market for health services.^16^ Finally, active engagement of academia and civil society can contribute to policy-making, implementation, and monitoring for health reforms: collaboration among these actors has the potential to exert collective pressure on governments and other stakeholders for promoting universality and equity in health policy.^27^

 The way these stakeholders inform the politics of reforms (“b” in Figure) depends on their specific interests and ideas: here interests refer to how the benefits of reforms are distributed among actors, whilst ideas concern the main values and ideologies inspiring their vision about policies. Interests and power distribution are traditionally intended as key factors in the sphere of politics. According to a more recent literature,^17,20^ ideological values are also important to consider in order to obtain a broader understanding of social protection reforms and the political discourse supporting these policies, which include also reforms to expand financial protection for health expenses.

 If we consider political negotiations about health financing in LMICs, both interests and ideas have considerable influence on the ensuing reforms. Ruling parties can expand social policies for financial protection against health expenditures as a means of legitimation to prevent the emergence of political opposition. Experiences in China,^19^ Rwanda,^17^ and Ethiopia^20^ are examples of regime legitimacy creation through the expansion of social insurance policies. The development of a comprehensive health insurance system can also be motivated by a desire to reduce financial dependency on donor contributions while increasing domestic resources, as in the case of Malawi.^28^ The incentive of political elections often underlies the decision to implement universalistic reforms in the health sector, or to revise agenda setting and policy formulation for this purpose. This has been the case for Thailand in 2001, Ghana in 2008, and Sierra Leone in 2010.^29^ In contrast, commercial interests and lobbying from multi-national companies boost policies in favour of the private health sub-sector.^30^

 Policy-making in the health system (“c” in Figure) requires coordinated action in multiple areas to be conducive for UHC.^19^ Health financing is key to ensure the system functions adequately.^6,31^ Mathauer and Carrin^32^ argue that two aspects of leadership and governance greatly affect achievements in terms of health coverage: first, the institutional design of rules for resources collection, resource pooling, and the purchasing of services; second, the organizational practice and capacity of the system to implement and comply with formal regulation. Moreover, leadership and governance aspects interact with health system inputs (such as infrastructure, human resources, equipment and supplies) to determine the policy outcomes. These aspects need to be interpreted through institutional lens: while “organisations” represent those actors who interact to influence health system and relevant reforms, “institutions” are the “rules of engagement between stakeholders” which crucially affect governance within health systems.^11^ In this sense, health system leadership is a key aspect to determine formal and informal institutions enabling organisations to learn, adapt, and interact in a constructive way to strengthen the health system.^13^ However, too often the institutional setting may foster inefficient behaviours among national stakeholders and, in turn, may alter patterns of engagement with international agencies.^11^

 This framework helps to disentangle the complexity of the political economy discourse about health financing reforms. To verify whether health reforms bring about advancement towards UHC (“d” in Figure), changes in the coverage dimensions of population, services, and costs are usually measured. These refer, respectively, to the proportion of the population that has financial protection, the range of services that are available, and the proportion of the costs of those services that are covered. Finally, it is expected that UHC, while increasing access to essential health services and improving financial protection, ultimately lead to better health outcomes for the population^12^ (“e” in Figure).

###  Historical Overview of Health Financing and Reforms

 Uganda presents a pluralistic system where service provision is divided among public and private sub-sectors.^33^ Within a decentralized architecture, districts are responsible for healthcare delivery, whilst the central government formulates policies and is responsible for supervision.^34^ The country constitutes a valid case study to examine the issue of healthcare financing; government expenditure as percentage of total health expenditure (THE) has been uneven over time^35^ and lower than that of neighbour countries such as Kenya and Tanzania.^36^ Currently, health spending indicators and the public budget for health are well below the recommended international targets, while sector financing is highly dependent on donor funding and direct payments^[1]^. The insurance sector is under-developed and contributes little to health financing^[2]^.^37^ As in many LMICs, impoverishing effects due to health costs are critical: for the 12% of the population, health expenditures represent more than 10% of total income.^38^ Out-of-pocket expenses still represent 42% of THE, and occurrence of financial catastrophes have not declined over the last two decades.^39,40^

 Over the last period of political stability, social protection policies in Uganda have exhibited specific features of political economy. As we focus on the last two decades, a recent analysis considers the year 2008 to distinguish among two distinct periods with respect to expenditure allocation criteria.^41^ The first period was characterised by high priority spending on social services in accordance with a national poverty reduction strategy. In the health sector, the principles of decentralisation, primary healthcare, health system strengthening, community participation and a sector wide approach constituted the chief reforms.^42^ At the global level, the increase in health funding was also encouraged by important initiatives such as the Heavily Indebted Poor Country Initiative for debt relief and Global Health Initiatives. Moreover, the international framework of the Millennium Development Goals provided further stimulus to the decision of regarding healthcare as a strategic priority for development.

 During the 2001 pre-elections phase, the President launched the “free healthcare” policy by abolishing user-fees in public facilities, thus helping to improve access to health services for the poor.^33^

 The second period of expenditure allocation began in 2010 and reflects a new development strategy firmly centred on the goal of achieving higher economic growth. The government’s decision to favour growth-enhancing sectors has involved a significant shift away from social spending and a greater support for infrastructure spending.^43^ At the same time, international actors emphasised the need to strengthen social policies.^44^ Public expenditure on health began to stagnate and efforts for decentralization, primary healthcare reforms, and public-private partnerships in health declined.^45^

 Over the last decade, efforts by the central government have not been adequate to strengthening the system for service delivery.^33^ Geographic accessibility continued to improve^[3],46^ but low domestic revenue flows and modest public budget allocations were not sufficient to meet demand for services.^39^ As a result, the quality of care in government facilities deteriorated, with frequent shortages of essential medicines and poor availability of human resources lowering effective coverage^[4]^.^46-48^ Given the evident financial weaknesses affecting the health system in recent years, the design of a public health insurance program has been a recurring theme of debate among national stakeholders.^49,50^ However, discussion of a possible National Health Insurance (NHI) scheme has been inconclusive for a long time,^51^ and the NHI Bill passed by Parliament only in March 2021.

## Methods

 The analysis draws on two main qualitative research methods, namely a desk review and KIIs with major players in the health sector both at the national and at the district level^[5]^. The review covers academic writings, policy documents, technical reports, and government policy briefs. We reviewed all available policy documents on the health sector produced in the sector by the central public authority for planning and policy-making going back to 1999, when the country started to develop guidelines for national health policy. Indeed, the position of the central government for health financing reforms is expressed in the core documents for planning and policy-making in the sector^[6]^. We consulted academic articles and books, as well as technical reports and background papers by other major stakeholders operating in the health system. Table 1 describes the main documents covered by the desk review (see Supplementary file 1 for the full list of consulted documents).

**Table 1:** Summary List of Consulted Documents for Desk Review

**Type of Document**	**Authors' Category as Stakeholders**	**Organisation Represented**	**No. of Documents (Total = 82)**
Official government documents	Government	Ministry of Finance Planning and Economic Development, Ministry of Health, Uganda Bureau of Statistics	25
Academic article, chapter or book	Development partners	World Health Organisation, Belgian Development Agency, Doctors with Africa CUAMM	23
	Academia	Academicians from Ugandan universities and foreign academic organisations, independent experts	
Working or discussion paper	Development partners	World Bank, WHO, UNICEF	18
	Academia	Makerere University, New York University, Manchester University, Ghent University	
	Civil society	Advocates Coalition for Development and Environment	
Report	Private sector	Ugandan Catholic Medical Bureau	16
	Development partners	International Monetary Fund, World Health Organisation, Belgian Development Agency, UK Department for International Development, US Agency for International Development	
	Academia	Makerere University, Economic Policy Research Centre, Birmingham University, Overseas Development Institute	
	Civil society	CORDAID, Global Network for Health Equity, Save for Health Uganda	

Abbreviations: WHO, World Health Organization; UNICEF, United Nations Children’s Fund.

 Individual interviews targeted firstly major stakeholders involved in health reforms and policy-making at the central level: KII participants were purposively selected based on their current or previous roles in the Ugandan health system. In total, we conducted 32 KIIs with national representatives of central government (including both technical and political leaders at the Ministry of Health), private sector and medical bureaus, academia, health development partners from bilateral cooperation and United Nations agencies, and civil society organisations. Furthermore, we performed 28 interviews in the district of Oyam^[7]^ with technical and political leaders, health providers of public and private facilities, Village Health Workers, and community leaders at the district level. Table 2 provides a summary list of the main stakeholders involved in the interviews^[8]^. Whilst most of these actors are the ones driving policy-making for reforms, the position of the general population is represented by the civil society and community leaders at the district level.

**Table 2:** Summary List of KIIs

**Level**	**Stakeholders**	**Organisations Represented**	**No. of Participants**
National	Government	Ministry of Health, National Planning Authority	6
	Private sector	Ugandan Catholic Medical Bureau, Pharmaceutical companies	4
	Development partners	World Bank, WHO, Belgian Development Agency, UK Department for International Development, US Agency for International Development, Doctors with Africa CUAMM	9
	Academia	Universities and independent experts	9
	Civil society	Save the Children, CORDAID, Save for Health Uganda	4
			**Total: 32**
District	Government	District Health Office, District Local Government	13
	Private sector	Ugandan Catholic Medical Bureau	2
	Development partners	Doctors with Africa CUAMM	1
	Civil society	Community leaders, village health workers	12
			**Total: 28**

Abbreviations: WHO, World Health Organization; KIIs, key informant interviews.

 Ethical issues were set using a protocol on high-level ethical standards and approved by the authors institutes. All respondents were asked to provide informed consent to participate in the study in respect of anonymity, and no ethical concerns arose during the research. Specifically, the informed consent presented assumptions and interests in the research topics by the investigators, as well as modalities of participation and treatment of data and contacts of investigators in case of questions or additional comments. Data collection took place during three missions in Uganda between November 2018 and January 2020, and interviews were performed in Kampala and in Oyam district within safe places (mainly offices and workplaces of participants) with no presence of external people.

 Interviews were conducted using semi-structured questionnaires that had been previously tested by the investigators to verify whether the contents of the political economy framework were clearly understood by participants. The topics covered by the national stakeholders’ questionnaire are the following: stakeholders’ function within the health system; major reforms and policies affecting health financing; the role of ideology and power differences in driving change; results in terms of health coverage; the current debates about UHC; and the main challenges and opportunities for enhancing financial coverage. These topics echo domains in our conceptual framework and enrich the discourse on political economy. The questionnaire used with district-level stakeholders was adapted to investigate access to health services and financial coverage for the population, thus focusing mainly on the sphere of coverage outcomes in the political economy framework. Interviews were conducted in English by one of the investigators^[9]^, audio recorded (with permission from participants), and then transcribed verbatim. Average duration of each interview was around 40 minutes. Documents and interview transcriptions were coded manually employing selective coding by identifying the central issue of healthcare financing as the core category of analysis; then we categorized other topics according to domains associated with our conceptual framework. The data relevant to each category was identified and analysed using a constant comparative method, in which single items are systematically checked with the rest of available information in order to triangulate findings and establish sound connections between categories.^52^ Moreover, the reporting of qualitative data collected through interviews follows consolidated criteria from the COREQ (COnsolidated criteria for REporting Qualitative research) checklist.^53^ While the desk review has been initially functional to inform the early stages of the investigation, depicting the historical overview of reforms, it was then used throughout the following phases of the investigation. Indeed, after concluding data collection, we systematically integrated evidence from the KIIs with the findings from the desk review.

 We acknowledge some methodological limitations to this study. First, given the great diversity of actors underlying the political economy negotiation some categories of stakeholders may be underrepresented in the sample of respondents. Second, although this does not hinder generalisability of the main findings, interviews at the local level were performed in one single district. Third, the historical path affecting the political discourse is analysed considering only the last two decades, since we decided to focus on the current implications of health financing reforms.

## Results

 The political discourse surrounding health financing in Uganda is animated by multiple actors and we analysed their position and role with respect to key reforms for improving financial protection such as the abolition of user-fees and the potential implementation of a NHI scheme. Following the line of reasoning illustrated in our conceptual framework, we present the main findings by referring to the domains of “stakeholder and institutions,” “politics,” and “policy” for health financing reforms as depicted in Figure (“a,” “b,” and “c” spheres). Findings from interviews at the district level shed more lights on the domain of “health coverage outcomes” (“d” sphere) and, specifically, financial protection for the general public.

###  Stakeholders’ Position in the Health Sector

 As we refer to the domain of stakeholders in Figure (“a”), five main categories of actors can be identified with respect to their role for health financing, namely government, private sector, development partners, academia, and civil society.

 The government, after user-fees abolition in 2001, only increased per capita health expenditure marginally, while public investments to enhance healthcare delivery have been inadequate.^33,36^ Starting from the second decade, according to several KIIs, central government did not provide clear guidance about health system reforms and services provision, although it is responsible for policy formulation.

 In recent years, the private for profit (PFP) sub-sector has expanded substantially. Low quality healthcare in public hospital and health centres has partly contributed to the higher utilization of PFP facilities by the population.^50^ However, the lack of common regulation of quality standards and pricing raises concerns about the fragmentation of the healthcare landscape.^33^ The collaboration between private and public organisations was less vibrant over the last decade, and financial contributions from the government to the private not for profit (PNFP) sector experienced a decline.^54^ In percentage terms, “the health budget provided to the PNFP sector increased slightly between 2000 (5.3%) and 2005 (8.5%) but gradually reduced to 2% in 2014.”^55^

 Considering the position of development partners with respect to health financing, poor accountability for large sums of money involving the Ministry of Health has led to important changes in the form of support for health initiatives.^56^ During the late 2000s, a shift occurred from budget support to vertical programs with poor coordination and weak system strengthening.^42^ Nonetheless, programmes and initiatives driven by development partners continue to play a central role for healthcare financing and services provision.^57^

 Looking at the role of other stakeholders, there is a consensus among many KIIs that the available evidence produced by academia does not currently influence the process of policy-making in the health sector to any real extent. Some informants argue that, in the foreseeable future, the development of strategic plans within the Sustainable Development Goals framework will make the role of academia more relevant^[10]^.

 KIIs indicated that civil society organisations also contribute to the evidence base on health sector practices and have repeatedly called for additional investment and effort to be directed towards healthcare. Although actors from the civil society often create partnerships with donors, governments and local communities,^58^ many respondents argue that support for specific initiatives do not translate into influential negotiation power to affect the overall process of decision making at the national level.

 Finally, we focus on the position of the community within the health financing system. A significant proportion of the population continues to bear a large financial burden for out-of-pocket health expenditures, which are likely to lead to disparities in access to quality health services.^36,59^ While involving the local community is vital for primary healthcare effectiveness and the achievement of UHC,^60^ several informants believe that the dominant approach is still oriented towards curative services and considers households as mere recipients of healthcare. As pointed out by a recent study,^58^ the general public is largely excluded from policy design and decision making at the district level.

###  Politics for Health Financing

Table 3 summarizes the main findings related to the politics sphere (“b” in Figure) for health financing reforms, highlighting differences in influence among actors and their respective contributions in terms of interests and values that shape policy outcomes.

**Table 3:** Stakeholders and Politics

**Stakeholders**	**Influence**	**Interests and Ideologies**	**Implications for Policy-Making**
Central government	Weak guidance for reforms and lack of political will to be the first player in the sector	Productive sectors and market expansion as strategic priorities	Poor leadership in the sector; expansion of health facilities infrastructures without proper functionality
Private sector	Strong economic power	Market supremacy	Development of PFP sub-sector without effective regulation
Development partners	Important financial contribution and influence	Fragmented preferences of single donors	Vertical programs without harmonization
Academia	Potential increasing influence in the negotiation process	Evidence-based approach	Not yet systematic use of evidence for policy design and policy-making
Civil society and population	Low influence in the negotiation process	Participatory bottom-up approach	No systematic engagement of civil society and population

Abbreviation: PFP, private for profit.

 The commitment of central government to the health sector has declined over the last decade, as demonstrated by the stagnant pattern of public health expenditure as a percentage of gross domestic product.^57^ A significant increase in competition within the political landscape and the change in leadership at the Ministry of Health may have contributed to a shift of national priorities from social services to productive sectors during the second decade.^54,61^ Most representatives of the central government expressed the idea that devoting efforts to infrastructure (such as roads and railway, but also physical infrastructures for healthcare provision) will lead to positive spill-over effects on health, since expansion of infrastructures is considered as an enabling condition to progress towards UHC^[11]^. In this sense, different priorities are not conceived as mere alternatives. A stakeholder from the Ministry of Health argues^[12]^:

*“The Ministry of Health is not the only responsible for health: social determinants of health are beyond this sector, and if we do not address social determinants many causes of diseases such as safe water, housing, personal behaviours are neglected. We believe that promoting a multisectoral approach will allow the country to record faster progress towards UHC.” *

 According to most respondents, there is a lack of consistent political commitment at central level to enforce and strengthen public health service delivery and, specifically, tension exists between the Ministry of Finance and the Ministry of Health concerning strategic policy-making for healthcare. Moreover, the Ministry of Gender Labour and Social Protection has the mandate to ensure social protection which is closely connected with the objective of enlarging financial protection for health expenses; according to several respondents, however, this ministry has much less contracting power than the other two. A representative of development partners explicitly states that the government has currently no interest in being the first player for the provision of health services and, thus, for healthcare financing. Consequently, development partners unanimously believe that health services have deliberately been delegated to them, who heavily finance the sector.

 Many key informants reiterate the common opinion that, over the last decade, much more scope than before has been given to market forces on the one hand, and to development partners on the other. Accordingly, a recent analysis of healthcare financing in the country attributes the drop in public funds to the crowding out effect of external subsidies.^36^ As expressed by a national academic, the presence of external donors creates a disincentive for central government to invest in the health sector^[13]^:

*“Maybe there is a side effect: as donors’ funds increase, government responsibility for health reduces, so you don’t see sufficient increase in the public budget as [it might be] expected.”*

 Several participants affirm that the presence of international donors is particularly important in specific areas, such as tackling Malaria, HIV and TB. The vulnerability of Uganda to fluctuations in development partners’ contributions is recognized in some studies.^36,50,62^ In this regard, a participant from a development partner organisation argues^[14]^:

*“Much of the budget for basic services is donors dependent [and] this means that the State is very vulnerable. If […] the Americans decide to leave the country, it would be a disaster. [This] is a risky situation.” *

 The private sector is also expanding its influence over services delivery. The strategic goal of promoting national economic growth is reflected in the health sector through renewed emphasis on market expansion.^35^ As a result, inequalities in access to services are increasing,^36^ while market forces tend to advantage those who are better placed to afford health. One independent expert declares^[15]^:

*“The shift […] is towards those who are economically powerful: the rich now have a greater voice in policies. […] Responding to investors in the sector, [and] responding to those who have money has become more important than having service coverage for those who need it most.” *

 Whilst the influence of the PFP and development partners for health financing and policy design is increasing, the relevance of civil society and general public for policy design is still minimal, as confirmed by a district-level stakeholder analysis.^58^ Similarly, many respondents observe that the current involvement of academia in the negotiation process does not translate into systematic use of evidence to inform reform processes. However, the SPEED (Supporting Policy Engagement for Evidence-Based Decisions) initiative which directly involves the universities into the definition of a roadmap for UHC in the country represents a factor of optimism for the future.

###  Implications for Policy Reforms 

 Values and interests of the most influential stakeholders have driven the negotiation process concerning policy design and implementation for healthcare financing (“c” in Figure) and, in particular, for the case of user-fees abolition and NHI discussion.

 In 2001, the President launched the “free healthcare” initiative as part of political discourse regarding key reforms. According to several analyses, the vision of universal access to basic healthcare was intended to legitimise the government during a period of transition to a multi-party system of governance.^54,63^ Similarly, many respondents argue that the ideological position of “free healthcare” was motivated by political gain of the elite who had interest to maintain the status quo in a landscape of increasing political competition.

 After the change in the government strategic vision, the dominant ideology became the supremacy of market forces. Meanwhile, discussions on the reform of NHI remained inconclusive for a long time with members of parliament who did not achieved agreement on the design of a possible scheme. A prepayment mechanism involving financial contributions from users would contradict the promise of “free healthcare” and, according to many KIIs, the President is apparently reluctant to implement this reform. The ambiguity between the “free healthcare” slogan from the government and the design of NHI remains thus crucial for health financing reforms and heavily influences the political decision-making process.

 Most informants from the central government suggest that the negotiations process for NHI is delayed due to the conflicting commercial interests of private companies, fearing a reduction of their market power, and basic misunderstandings of insurance principles by formal sector employees, who interpret membership to insurance as a reduction in their salary. In general, public policies designed by the ruling party in the country are often designed to retain support from prominent factions.^61^ In the case of NHI, political incentives are provided by private companies and trade unions to refrain from implementing a comprehensive scheme covering the whole population.^49^ On one side, the private sector fears competition between social health insurance and commercial schemes; on the other side, trade unions are concerned about payroll deduction from workers’ pay. Furthermore, after corruption scandals in the public sector, these actors have doubt about the government capacity and transparency in implementing a unique national scheme. The process to design the NHI scheme failed to create ownership among the main players in the private sectors and the lack of backing from these stakeholders protracted the discussion.^49^ In other words, poor stakeholder’s engagement appears to be a critical factor both for the decision of user-fees abolition, which has not been discussed within a health secotr forum, and for the ongoing debate about NHI.

 Overall, conflicting interests, ideas, and perceptions about insurance do not create favourable ground for cultivating a consensus on the design and implementation of a national insurance to improve financial protection. Including the informal sector within the health financing system represents a relevant issue. Although participants from civil society and the PNFP sub-sector have less voice than other stakeholders, they advocate active involvement of the community within the health system. The idea of financing healthcare in a sustainable way and, meanwhile, empowering the demand side is reflected in the design of Community Based Health Insurance.^64^ This model aims to provide financial protection to individuals in the informal sector. Interest in Community Based Health Insurance is increasing in Uganda, but the implementation of single schemes remains highly fragmented in the absence of an overall public insurance programme at the national level.^65^

 In conclusion, the current political negotiation process for health financing reforms is failing to harmonize interventions driven by individual stakeholders: development partners are mainly financing vertical programs, whilst the public sector, PNFP and PFP sub-sectors are not yet coordinated to contribute to a unique system for resource collection, pooling and services provision. In other words, both institutional design and organizational practices to guarantee the adequate functioning of the system are not yet favourable for expanding financial coverage in an equitable manner.

###  Consequences for Coverage Outcomes and Financial Protection

 Given the lack of comprehensive and equitable health financing reforms at the national level, outcomes in terms of population, services and costs coverage (“d” in Figure) are not improving. As we consider a rural and informal setting (Oyam district), the political economy discourse results into a generally low level of financial protection at the local level The vast majority of the population lacks adequate protection for health expenses. Most interviewees in the district argue that impoverishing effects due to health expenditures are becoming more frequent over recent years as the private sector expands without adequate regulation and the public sector is not able to offer adequate quality of care^[16]^:

*“The main concerns about accessing healthcare are, on one side, the poor availability of drugs and medicines in public health centres and, on the other side, the [lack of] affordability of services in private clinics.” *

 Indeed, most services which are supposed to be guaranteed at the public facilities are not provided in practice, whilst private health facilities are not affordable to many families. Given such difficulties, some community representatives observe that the spirit of solidarity among the population in rural area is high, and the practice of risk-sharing for health expenditures is quite widespread^[17]^:

*“Community members use to support each other during illness, providing in-kind and monetary gifts. This spirit is stronger in remote areas where utilising health services is really challenging.” *

 Health providers stated that sometimes community groups bring their pooled contributions to pay user-fees for admitted members. However, evidence from a specific study in Uganda^66^ shows that the absence of a coherent policy framework prevents these informal mechanisms from operating as a functional scheme of social protection. Furthermore, some authors^65^ pointed out that the poorest remain excluded from this informal safety-net since they cannot afford to join community groups. The fact that solidarity regards only members of defined groups implies an important equity concern, since risk-sharing practices bring advantages only for those who share a common identity. Consequently, caution is needed when considering the potential role of these informal practices for health financing: spontaneous initiatives by the population require to be channelled through a solid legislative framework in order to effectively contribute towards a comprehensive scheme of financial protection.

 We can interpret the rationale to rely on informal networks for coping with health expenditures as partly due to the delay to implement effective national reforms for financial protection. Indeed, the population is not supported neither by the government nor by the private sector to improve coverage outcomes.

## Discussion and Conclusion

 The interpretation of the main findings through the developed framework allows us to disentangle the dynamic and incremental processes of political economy for health financing reforms in Uganda, and to interpret the current level of financial protection for the population. Whilst transition towards UHC requires “*several essential forces […] to mature and come together,*”^19^ we contend that political conditions are currently delaying an effective expansion of financial protection coverage for the population.

 The negotiation process for health financing reforms is characterised by divergent ideologies concerning healthcare as well as conflicting interests for the main stakeholders’ categories. In recent years, central government has not regarded social services and, in particular, healthcare, as a strategic priority, and the ensuing public budget remained stagnant. On the other hand, development partners and private organisations are gaining influence within the sector, but they lack coordination. In contrast, academics and civil society have at the moment weaker voices within the national debate on health financing.

 The dominant ideology of market supremacy and the regime’s strategic vision to transform Uganda into a middle-income economy has added to an unfavourable background for designing and implementing a comprehensive insurance scheme. The dichotomy between the slogan of “free healthcare” and the planned reform of NHI has not been totally solved, and engagement of important stakeholders into the process of reform design has been low. Consequently, a broad consensus about a comprehensive scheme for financial protection has not yet been reached, and an extremely fragmented and inequitable landscape for health financing remains in place with weaknesses in terms of service delivery and harmonization of interventions undermining the capacity of the system to improve coverage outcomes.

 Further to delaying conditions, the political economy analysis permitted to identify two enabling factors that provide positive stimulus for advancing the negotiation process behind health financing reforms. Whilst the scope of this political process is national, the two factors originate, respectively, from the international arena and from the local community background. First, the 2030 Agenda is creating strong momentum towards UHC that can be exploited at the national level to unlock the negotiation process for a comprehensive scheme of financial protection. The mission to promote a broad access to essential health services without suffering financial hardship needs to be translated into national-level reforms for health financing: in this sense, the global community can exert pressure on the central government in order to clarify the ambiguity between the “free healthcare” policy and the debate on NHI. The political process to define the national strategy for the goal of UHC also constitutes the opportunity to create an effective platform of dialogue and discussion between national partners from different sectors (such as the private sector and the academia). Second, another stimulus comes from bottom-up leverage involving the population and, in particular, the informal sector through community-based initiatives aimed to expand the practice of risk-sharing for health expenditures. Increasing collection and pooling of prepayment contributions and promoting an active role in the health system for linking the demand and supply-side of healthcare represents a promising opportunity to expand financial coverage; however, this architecture for health financing can be sustainable and efficient only if coordinated by a multi-level governance.^67^ In other words, if efforts by the community represent an important boost, poor stewardship by the government does not permit to effectively advance towards UHC. Engagement of the civil society and the general public can bring important advantages to health system strengthening, but this requires a clear political will and does not imply a shift of responsibility away from the central government.

 To conclude, this analysis contributes to the emerging literature on the political economy of health sector reforms in LMICs. The study highlights key political factors that influence the context-dependent trajectory of Uganda for health financing reforms aimed at improving financial protection for the population. On one side, at the national level, poor stewardship of the central government into the health sector and lack of effective platforms of dialogue involving different stakeholders prevent to achieve the effective implementation of a comprehensive scheme for financial protection. On the other side, both the global agenda focused on the overarching goal of UHC and spontaneous bottom-up initiatives at the local level to improve health coverage can constitute opportunities to weight on the reactivity of the system to develop a clear policy agenda for health financing and financial protection. The adoption of a political economy perspective is relevant to enhance the understanding on the main processes shaping progress towards UHC and the usefulness of applying this analytical lens goes beyond the single case study of Uganda. It will be important, thus, to utilise political economy frameworks such as the one presented here as key to interpret the experiences of different LMICs.

## Acknowledgements

 We are grateful for the logistical support of Doctors with Africa CUAMM in Uganda. Earlier drafts of the paper strongly benefitted from comments by David A. Clark and Gavino Maciocco. We wish to thank all the participants to KIIs for their precious contribution to the research.

## Ethical issues

 Ethical issues were set using a protocol on high-level ethical standards and approved the Ethic Committee for Research of the University of Florence and by Doctors with Africa CUAMM. All respondents were asked to provide informed consent to participate in the study, and no ethical concerns arose during the research.

## Competing interests

 Authors declare that they have no competing interests.

## Authors’ contributions

 MN and MB conceived and designed the study, analysed the data, wrote the draft manuscript, and participated in the interpretation of the findings and revision of the manuscript. GP contributed to the implementation of the study, participated in the data analysis, interpretation of the findings and revision of the manuscript. All authors read and approved the final version of the manuscript.

## Funding

 Doctors with Africa CUAMM provided us with logistical support during the field research in Uganda. No other forms of support/funding were received.

## Supplementary files

Supplementary file 1. List of Consulted Documents for the Desk Analysis.

## Endnotes

 [1] On average, about 8% of public spending was devoted to the health sector between 2012/2013 and 2016/2017. This is well below the Abuja declaration target of 15.^57^ During the same period, the total health budget as a percentage of gross domestic product has remained about 1% compared to a regional average of 1.9 for Sub-Saharan African countries and the international target of 5% for LMICs.^36^ On a per capita basis, between 2012/2013 and 2016/2017 the government spent US $8 on health, against the WHO target of $34.^36^ [2] Figures from the Ministry of Health^57^ show that 42% and 43% of THE respectively were covered by development partners and private funds in 2015/2016. In contrast, the public sector contribution only accounted for 15% of THE. Overall, health expenditure per capita in 2017 was US $51, against the minimum of US $84 recommended by the WHO.^57^ [3] The proportion of households living within a radius of 5 km from health facilities raised from 72 in 2010/2011 to 86% in 2016/2017.^68,46^ [4] In 2013/2014, only 45% of health centres of fourth level (IV) have been found to be functional in terms of availability of Comprehensive Emergency Obstetric Care services.^69^ The density of health workforce, which increased from 0.498 in 2011/2012 to 0.710 in 2014, remains well below the WHO recommended target of 2.28 health workers per 1000 people.^47^ [5] In order to identify the scope of priority setting for healthcare, finalize the list of key informants and design interview questions, we performed a preliminary research phase by participating to eight workshops with health sector stakeholders at the district level and to two national conferences on UHC. [6] National Health Policy I and II, National Budget Framework Papers, Health Sector Strategic Plans, Health Sector Development Plans, and National Health Accounts. [7] The regional health system in Oyam is similar to the rest of Uganda, featuring a wide variety of health providers. Due to a long post-conflict period, this district records lower coverage outcomes for health services than those at the national level,^70^ and financial obstacles to healthcare utilisation are still critical for the local population.^67^ Therefore, the district constitutes a solid case study with analytical relevance regarding financial protection for the population. [8] Such categorization does not reflect uniform ideological positions and influence in the negotiation process. [9] This person, with a PhD in Development Economics, had already previous experience of research work in Uganda and a basis of knowledge about the national context. [10] Indeed, several universities and research entities have been engaged in producing a country-specific roadmap towards UHC to orient policies for the health system.^71^ [11] For example, geographic accessibility to health services improved after great efforts to build new health facilities. [12] KII, Kampala, February 25, 2019. [13] KII, Kampala, February 22, 2019. [14] KII, Kampala, January 30, 2019. [15] KII, Kampala, February 27, 2019. [16] KII, Oyam, January 21, 2020. [17] KII, Oyam, January 23, 2020.
