# Supplementary material for: Health Coverage and Financial Protection in Uganda: A Political Economy Perspective
Source: Int J Health Policy Manag. 2021 Aug 29;11(9):1894–904. doi: 10.34172/ijhpm.2021.116 (PMC9808243; doi:10.34172/ijhpm.2021.116)
Supplement: Supplementary file 1 — List of Consulted Documents for the Desk Analysis. [file ijhpm-11-1894-s001.pdf]

Article title: Health Coverage and Financial Protection in Uganda: A Political Economy Perspective

Journal name: International Journal of Health Policy and Management (IJHPM)

Authors' information: Maria Nannini[1]*, Mario Biggeri[1], Giovanni Putoto[2]

[1]Department of Economics and Management, University of Florence, Florence, Italy.

[2]Doctors with Africa CUAMM, Padova, Italy.

(*corresponding author: maria.nannini@unifi.it)

Supplementary file 1. List of Consulted Documents for the Desk Analysis

Official government documents:

1. Ministry of Finance Planning and Economic Development (MoFPED). 2012. "Central Government Public Expenditure and Financial Accountability Assessment". Government of Uganda, Kampala.
2. MoFPED. 2018. "Background to the Budget Fiscal Year 2018/19". Government of Uganda, Kampala.
3. MoFPED. 2018. "National Budget Framework Paper FY 2019/20-Fy 2023/24". Government of Uganda, Kampala.
4. Ministry of Health (MoH). 1999. "National Health Policy I." Government of Uganda, Kampala.
5. MoH. 2009. "Uganda Health Accounts 2008/09 – 2009/10". Government of Uganda, Kampala.
6. MoH. 2010. "Health Sector Strategic & Investment Plan 2010/11 – 2014/15". Government of Uganda, Kampala.
7. MoH. 2010. "National Health Policy II: Promoting People's Health to Enhance Socio-economic Development". Government of Uganda, Kampala.
8. MoH. 2011. "Uganda Health Accounts 2010/11 – 2011/12". Government of Uganda, Kampala.
9. MoH. 2012. "Annual Health Sector Performance Report 2011/12". Government of Uganda, Kampala.
10. MoH. 2013. "Annual Health Sector Performance Report 2012/13". Government of Uganda, Kampala.
11. MoH. 2013. "Uganda Health Accounts 2012/13 – 2013/14". Government of Uganda, Kampala.
12. MoH. 2014. "Annual Health Sector Performance Report 2013/14". Government of Uganda, Kampala.
13. MoH. 2015. "Annual Health Sector Performance Report 2014/15". Government of Uganda, Kampala.
14. MoH. 2015. "Health Sector Strategic & Investment Plan 2015/16 – 2019/20". Government of Uganda, Kampala.
15. MoH. 2015. "Uganda Health Accounts 2014/15 – 2015/16". Government of Uganda, Kampala.
16. MoH. 2016. "Annual Health Sector Performance Report 2015/16". Government of Uganda, Kampala.
17. MoH. 2016. "Health Financing Strategy 2015/16 – 2024/25". Government of Uganda, Kampala.
18. MoH. 2017. "Uganda Health Accounts 2016/17 – 2017/18". Government of Uganda, Kampala.
19. MoH. 2017. "Annual Health Sector Performance Report 2016/17". Government of Uganda, Kampala.
20. MoH. 2018. "Annual Health Sector Performance Report 2017/18". Government of Uganda, Kampala.
21. MoH. 2019. "Booklet on the Planned National Health Insurance Scheme". Government of Uganda, Kampala.
22. Uganda Bureau of Statistics (UBOS). 2015. "National Service Delivery Survey 2015". Government of Uganda, Kampala.
23. UBOS. 2017. "National Population and Housing Census 2014 Area Specific Profiles Oyam District". Government of Uganda, Kampala.
24. UBOS. 2017. "Uganda National Household Survey 2016". Government of Uganda, Kampala.
25. UBOS & ICF. 2018. "Demographic and Health Survey 2016". Government of Uganda, Kampala.

Academic articles, chapters, and books:

1. Awor P, Bisase H, Lochoro P, Curtale F. 2018. "How is the Partnership between the Public and Non-Public Sectors Evolving to Strengthen Universal Health Coverage?" Chapter within the Book "*Universal Health Coverage UGANDA"*. pp. 1-17.
2. Baine SO, Kakama A, Mugume M. 2018. "Development of the Kisiizi hospital health insurance scheme: lessons learned and implications for universal health coverage". *BMC Health Serv Res*. 18: 455, 1-9.
3. Barya J-J. 2011. "Social Security and Social Protection in the East African Community". *Fountain Publishers*, Kampala. http://www.kituochakatiba.org/sites/default/files/publications/Social Security and Social Protection in the East African Community.pdf.
4. Basaza RK, Criel B, Van Der Stuyft P. 2010. "Community health insurance amidst abolition of user fees in Uganda: The view from policy makers and health service managers". *BMC Health Serv Res*. 10:33, pp 1-10. doi:10.1186/1472-6963-10-33
5. Basaza RK, O'Connell TS, Chapcakova I. 2013. "Players and processes behind the national health insurance scheme: a case study of Uganda". *BMC Health Serv Res*. 13:357, pp. 1-13. doi:10.1186/1472-6963-13-357
6. Ekirapa-Kiracho E, Paina L, Kananura RM, et al. 2017. "Nurture the sprouting bud; do not uproot it. Using saving groups to save for maternal and newborn health: Lessons from rural Eastern Uganda". *Glob Health Action*. 10(4), pp. 92-102. doi:10.1080/16549716.2017.1347311
7. Habraken R, Schulpen L, Hoebink P. 2017. "Putting promises into practice: The New Aid Architecture in Uganda". *Dev Policy Rev*. 35(6):779-795. doi:10.1111/dpr.12294
8. Kjær AM. 2015. "Political Settlements and Productive Sector Policies: Understanding Sector Differences in Uganda". *World Dev*. 68:230-241. doi:10.1016/j.worlddev.2014.12.004
9. Kwesiga B, Zikusooka CM, Ataguba JE. 2015. "Assessing catastrophic and impoverishing effects of health care payments in Uganda". *BMC Health Serv Res*. 15(1), pp. 1-6. doi:10.1186/s12913-015-0682-x
10. Maniple E. 2009. "Why are donors running away from health in Uganda?" *Editorial Heal Policy Dev*. 7(3).
11. Mugisha J. 2004. "Reduction of user fees in the Private not for Profit Hospitals in Uganda: Implications for equity and sustainability". *Heal Policy Dev*. 2(3): 209-216.
12. Nabyonga Orem J, Zikusooka C. 2010. "Health financing reform in Uganda: How equitable is the proposed National Health Insurance scheme?" *Int J Equity Health*. 9(1):23. doi:10.1186/1475-9276-9-23
13. Nabyonga-Orem J, Mugisha F, Kirunga C, Macq J, Criel B. 2011. "Abolition of user fees : the Uganda paradox". *Heal Policy Plan*. 26:41-51. doi:10.1093/heapol/czr065
14. Okuonzi SAMA, Macrae J. 1995. "Whose policy is it anyway? International and national influences on health policy development in Uganda". *Heal Policy Plan*. 10(2):122-132.
15. Razavi SD, Kapiriri L, Abelson J, Wilson M. 2019. "Who is in and who is out? A qualitative analysis of stakeholder participation in priority setting for health in three districts in Uganda". *Heal Policy Plan*. 1-12. doi:10.1093/heapol/czz049
16. Ruhweza M, Baine S, Onama V, Basaza V, Pariyo G. 2009. "Financial risks associated with healthcare consumption in Jinja , Uganda". *Afr Health Sci*. 9(2): S86-S89.
17. Ssengooba F, Hongoro C. 2017. "Framing the Determinants of Health and Well- Being for Universal Health Coverage". In: *Universal Health Coverage in Uganda: Looking Back and Forward to Speed up the Progress*. Makerere University, Kampala. pp. 3-23.
18. Ssengooba F. 2017. "The Road Map to Universal Health Coverage". In: *Universal Health Coverage in Uganda: Looking Back and Forward to Speed up the Progress*. Makerere University, Kampala. pp. 413-433.
19. Ssennyonjo A, Namakula J, Kasyaba R, Orach S, Bennett S, Ssengooba F. 2018. "Government resource contributions to the private-not-for-profit sector in Uganda: evolution, adaptations and implications for universal health coverage". *Int J Equity Health*. 17(1):130. doi:10.1186/s12939-018-0843-8
20. Stierman E, Ssengooba F, Bennett S. 2013. "Aid alignment: A longer term lens on trends in development assistance for health in Uganda". *Global Health*. 9(1):1-11. doi:10.1186/1744-8603-9-7
21. Tashobya CK, Ssengooba F, Cruz VO. 2006. "Health Systems Reforms in Uganda: processes and outputs". Institute of Public Health, Makerere University, Kampala.
22. Xu K, Evans DB, Kadama P, et al. 2006. "Understanding the impact of eliminating user fees : Utilization and catastrophic health expenditures in Uganda". *Soc Sci Med*. 62:866-876. doi:10.1016/j.socscimed.2005.07.004

23. Zikusooka CM, Kyomuhang R, Orem JN, Tumwine M. 2009. "Is health care financing in Uganda equitable?" *Afr Health Sci.* 9 Suppl 2(2): S52-8.

Working and discussion papers:

1. Bukenya B, Muhumuza W. 2017. "The Politics of Core Public Sector Reform in Uganda: Behind the Façade". ESID Working Paper N. 85, Manchester University, UK.
2. Golooba-mutebi F, Hickey S. 2013. "Investigating the Links between Political Settlements and Inclusive Development in Uganda: Towards a Research Agenda". ESID Working Paper N. 85, Manchester University, UK.
3. Hickey S, Bukenya B, Izama A, Kizito W. 2015. "The Political Settlement and Oil in Uganda". ESID Working Paper N. 48, Manchester University, UK.
4. Hickey S, Bukenye B. 2016. "The Politics of Promoting Social Cash Transfers in Uganda". ESID Working Paper N. 69, Manchester University, UK.
5. Kasirye I, Ssewanyana S, Nabyonga J, Lawson D. 2004. "Demand for Health Care Services in Uganda: Implications for Poverty Reduction". MPRA Paper No. 8558. Makerere University, Kampala.
6. Kjær AM, Muwanga NK. 2016. "Inclusion as Political Mobilisation: The Political Economy of Quality Education Initiatives in Uganda". ESID Working Paper N. 65, Manchester University, UK.
7. Lukwago, D., 2016. "Health Spending in Uganda: Implications on the National Minimum Health Care Package". ACODE Policy Briefing Paper N.32. Kampala.
8. Okwero P, Tandon A, Sparkes S, McLaughlin J, Hoogeveen JG. 2010. "Fiscal Space for Health in Uganda". WB Working Paper N. 186. Africa Human Development Series. Washington, D.C. doi:10.1596/978-0-8213-8290-5 WP WB
9. OXFAM. 2013. "Universal Health Coverage: Why health insurance schemes are leaving the poor behind", OXFAM Briefing Paper N. 176. https://doi.org/10.4335/11.3.687-708
10. Smoke P. 2013. "Political Economy of Institutions for Service Delivery, Uganda Public Expenditure Review on Decentralization and Service Delivery Background". Background Paper N. 1. New York University.
11. Steurs L. 2018. "European Aid and Health System Strengthening: an Analysis of donor approaches in the DRC, Ethiopia, Uganda, Mozambique and the Global Fund". PhD Dissertation, Ghent University.
12. UNICEF. 2019. "Cross-Cutting Budget Issues Papers to Assist Strengthening Sector Dialogue and Budgeting". Kampala.
13. UNICEF Uganda. 2018. "Uganda: Political Economy Analysis". Kampala.
14. UNICEF Uganda. 2019. "Education Budget Issues Paper". Kampala.
15. UNICEF Uganda. 2019. "Health Budget Issues Paper". Kampala.
16. UNICEF Uganda. 2019. "Social Development and Child Protection Budget Issues Paper". Kampala.
17. UNICEF Uganda. 2019. "WASH Budget Issues Paper". Kampala.
18. Xu K, Evans DB, Kadama P, Nabyonga J, Ogwang Ogwal P, Mylena Aguilar A. 2005. "The Elimination of User Fees in Uganda: Impact on Utilization and Catastrophic Health Expenditures". Discussion Paper N. 4, Department Health System Financing, WHO, Geneva.

Reports:

1. Curtale F. 2015. "An overview of the health sector in Uganda". BTC Uganda, Kampala.
2. Development Pathways, DFID. 2012 "Uganda Social Protection Public Expenditure Review", Expanding Social Protection Programme, Government of Uganda, Kampala.
3. Djkstra, G. 2011. "Aid and Health Sector Performance in Uganda. Uganda" CORDAID, The Nederlands.
4. IMF, 2017. "Uganda: Selected Issues" Country Report No. 17/207, Washington, D.C.

5. Lister S, Baryabanoha W, Steffensen J, Williamson T. 2006. “Evaluation of General Budget Support” Uganda Country Report, International Development Department School of Public Policy, University of Birmingham, UK.
6. Odokonyero T, Mwesigye F, Adong A, Mbowa S. 2017. “Universal Health Coverage in Uganda: The Critical Health Infrastructure, Healthcare Coverage and Equity”. EPRC Research Series N. 136, SPEED Programme, Makerere University, Kampala.
7. Orach SO. 2014. “The Contribution of Religious Health Networks in Systems Strengthening through Innovations in Community Health Financing – The Case of Community Health Insurance in Uganda – Successes and Challenges”. Uganda Catholic Medical Bureau, Kampala.
8. Save for Health Uganda. 2017. “Second National Community Health Financing Conference Report”. Kampala.
9. Save for Health Uganda. 2018. “Third National Community Health Financing Conference Report”. Kampala.
10. Save for Health Uganda. 2019. “Fourth National Community Health Financing Conference Report”. Kampala.
11. Soors W, Devadasan N. 2010. “Community Health Insurance and Universal Coverage: Multiple Paths, Many Rivers to Cross”. World Health Report, Background Paper N. 48. WHO, Geneva.
12. Makerere University. 2017. “Symposium on Health Financing for UHC in LMICs” Proceedings Report, SPEED Programme, Kampala.
13. USAID, Ministry of Health Uganda. 2016. “Financing for Inclusive and Sustainable Health Services: Uganda Health Public Expenditure Review 2013/14-2016/17”. Kampala.
14. USAID, Ministry of Health Uganda. 2017. “Uganda Health Sector Budget Execution Bottlenecks Report”. Kampala.
15. Williamson T, Davies F, Aziz I, Hedger E. 2016. “Budget Support to Uganda 1998- 2012”. ODI, London, UK.
16. Zikusooka CM, Kwesiga B, Logony S, Abewe C. 2014. “Universal Health Coverage Assessment Uganda”. Global Network for Health Equity. doi:10.13140/RG.2.1.1174.7683
